# Strategies to guide the successful implementation of deprescribing in community practice: Lessons learned from the front line

**DOI:** 10.1177/17151635241240737

**Published:** 2024-04-13

**Authors:** Justin P. Turner, Kelda Newport, Aisling M. McEvoy, Tara Smith, Cara Tannenbaum, Deborah V. Kelly

**Affiliations:** Centre for Medicines Use and Safety, Memorial University of Newfoundland, Newfoundland and Labrador; Faculty of Pharmacy and Pharmaceutical Sciences, Monash University, Victoria, Australia; the Faculty of Pharmacy, Memorial University of Newfoundland, Newfoundland and Labrador; Université de Montréal, Québec; the Centre de recherche, Institut universitaire de gériatrie de Montréal, Memorial University of Newfoundland, Newfoundland and Labrador; Québec; the Faculté de Pharmacie, Memorial University of Newfoundland, Newfoundland and Labrador; Laval Université, Québec; and the School of Pharmacy, Memorial University of Newfoundland, Newfoundland and Labrador; Centre for Medicines Use and Safety, Memorial University of Newfoundland, Newfoundland and Labrador; Laval Université, Québec; and the School of Pharmacy, Memorial University of Newfoundland, Newfoundland and Labrador; Faculty of Pharmacy and Pharmaceutical Sciences, Monash University, Victoria, Australia; the Faculty of Pharmacy and Medicine, Memorial University of Newfoundland, Newfoundland and Labrador; Université de Montréal, Québec; the Centre de recherche, Institut universitaire de gériatrie de Montréal, Memorial University of Newfoundland, Newfoundland and Labrador; Laval Université, Québec; and the School of Pharmacy, Memorial University of Newfoundland, Newfoundland and Labrador

## Abstract

**Background:**

Sustainable implementation of new professional services into clinical practice can be difficult. In 2019, a population-wide initiative called SaferMedsNL was implemented across the province of Newfoundland and Labrador (NL), to promote appropriate medication use. Two evidence-based interventions were adapted to the context of NL to promote deprescribing of proton pump inhibitors and sedatives. The objective of this study was to identify and prioritize which actions supported the implementation of deprescribing in community practice for pharmacists, physicians and nurse practitioners across the province.

**Methods:**

Community pharmacists, physicians and nurse practitioners were invited to participate in virtual focus groups. Nominal Group Technique was used to elicit responses to the question: “What actions support the implementation of deprescribing into the daily workflow of your practice?” Participants prioritized actions within each group while thematic analysis permitted comparison across groups.

**Results:**

Five focus groups were held in fall 2020 involving pharmacists (*n* = 11), physicians (*n* = 7) and nurse practitioners (*n* = 4). Participants worked in rural (*n* = 10) and urban (*n* = 12) settings. The different groups agreed on what the top 5 actions were, with the top 5 receiving 68% of the scores: (1) providing patient education, (2) allocating time and resources, (3) building interprofessional collaboration and communication, (4) fostering patient relationships and (5) aligning with public awareness strategies.

**Conclusion:**

Pharmacists, physicians and nurse practitioners identified similar actions that supported implementing evidence-based deprescribing into routine clinical practice. Sharing these strategies may help others embed deprescribing into daily practice and assist the uptake of medication appropriateness initiatives by front-line providers. *Can Pharm J (Ott)* 2024;157:xx-xx.

**Knowledge into Practice**

- Implementation of evidence-based deprescribing initiatives in community practice can be challenging.
- Understanding the actions that facilitate the implementation of deprescribing and medication appropriateness initiatives in community practice from a multidisciplinary perspective is important for promoting collaborative practice change.
- Pharmacists, physicians and nurse practitioners identified 5 key actions that participants voted as most important for implementing deprescribing into their day-to-day practice.
- Identifying and sharing actions that support deprescribing in community practice may help other front-line health care providers sustainably implement deprescribing in their day-to-day practice.

**Mise en pratique des connaissances**

- La mise en œuvre d’initiatives de déprescription fondées sur des données probantes dans la pratique communautaire peut être difficile.
- Il est important de comprendre les mesures qui facilitent la mise en œuvre des initiatives de déprescription et d’usage approprié des médicaments dans la pratique communautaire d’un point de vue multidisciplinaire pour promouvoir le changement de pratique collaborative.
- Les pharmaciens, les médecins et les infirmières et infirmiers praticiens ont identifié 5 mesures clés choisies par les participants comme les plus importantes pour la mise en œuvre de la déprescription dans leur pratique quotidienne.
- L’identification et le partage des mesures qui soutiennent la déprescription dans la pratique communautaire peuvent aider d’autres fournisseurs de soins de santé de première ligne à mettre en œuvre de manière durable la déprescription dans leur pratique quotidienne.

## Introduction

In Canada, potentially inappropriate medications continue to be prescribed at a high volume despite offering low benefit and increased risk of harm.^1,2^ Consequently, efforts to promote the appropriate use of medications have become a priority at federal, provincial and territorial levels.^3^ Deprescribing is the supervised process of withdrawing an inappropriate medication with the goal of managing polypharmacy and improving outcomes.^4^ With deprescribing showing patient and system-level benefits,^5,6^ different policy strategies have been used internationally in an attempt to reduce inappropriate medication use.^7-10^ Such interventions have produced varied success (e.g., driver’s license restrictions in Denmark reduced benzodiazepine use),^11^ while some have produced unintended consequences (e.g., alprazolam rescheduling in Australia reduced use but increased mortality).^7,8,12-15^

The province of Newfoundland and Labrador (NL) has one of the highest prevalences of potentially inappropriate medications within Canada.^2^ Proton pump inhibitors (PPIs), which are often used long term unnecessarily,^16^ are used by 27% of the province, and sedatives, which can increase the risk of medication-related harm, are used long term by 22% of the population.^3^ The prevalence of these medications continued to increase after 2011^3^; therefore, in response to this pressing problem, in 2019, the NL government sought to scale up and spread evidence-based interventions to reduce the long-term use of sedatives and PPIs.^17^ This led to the development of SaferMedsNL (www.SaferMedsNL.ca), a theory-driven, evidence-based,^18,19^ province-wide initiative to promote safe medication use through deprescribing.

Although deprescribing may be considered an inherent component of good prescribing practice,^20^ clinicians often find it difficult to deprescribe in practice. Barriers to deprescribing have been reported by health care providers, including poor communication between health care providers and patients and lack of awareness, education and evidence-based tools.^21-27^ However, clear descriptions of the actions undertaken by front-line health care providers to overcome these barriers and deliver evidence-based deprescribing in clinical practice are not as well described.

Translating, adapting and implementing existing knowledge and clinical decision-making deprescribing tools into real-world practice has been identified as a logical next step in deprescribing research.^28^ Understanding which actions are used by front-line health care providers who are engaged in deprescribing in everyday practice is a key perspective in the implementation process, so that “what works” can be supported to optimize medication use from a multidisciplinary approach.

## Aim

The objective of this study was to identify and prioritize which actions supported the implementation of deprescribing in community practice for pharmacists, physicians and nurse practitioners across NL.

## Methods

### Setting

This study investigates the positive actions taken by health care providers to integrate deprescribing into their day-to-day practice as a result of the SaferMedsNL initiative, which began in 2019. SaferMedsNL adapted, scaled and implemented 2 successful evidence-based interventions to promote deprescribing: (1) a public awareness campaign coupled with health care provider education^18^ and (2) direct patient education by pharmacists, physicians and nurse practitioners.^19^ The SaferMedsNL public awareness campaign was run on TV, radio, social media and online. The campaign also ran in print, publishing articles in newspapers, advertising on the back of buses and printing posters for health care providers to use. These activities were supported with health care provider education. Direct-to-patient education was facilitated by adapting the D-PRESCRIBE trial.^18^ New funding supported pharmacists initiating conversations about appropriate medication use with long-term users of PPIs or sedatives and sending an evidence-based pharmaceutical opinion to the patient’s family physician if deprescribing was appropriate (pharmacies received $23 per consult).^19,29^ An additional fee of $10 was reimbursed if a follow-up conversation was had with the patient within 6 months to support the deprescribing process. Additional funding was not allocated for physicians or nurse practitioners. Health care providers were able to order free patient educational brochures and promotional posters to support the provision of direct patient education.

### Study design and sample

Community pharmacists, family physicians and nurse practitioners from across NL were invited to participate in a 2-hour virtual focus group via Zoom. Invitations were distributed through SaferMedsNL stakeholders, the Pharmacists’ Association of Newfoundland and Labrador, the Newfoundland and Labrador Medical Association and the College of Registered Nurses of Newfoundland and Labrador, as well as being posted on Twitter (@SaferMedsNL). This study aimed to recruit approximately 10 pharmacists and 10 prescribers (physicians and nurse practitioners) using a purposive sampling approach that sought to produce variation across provider types (physicians or nurse practitioners and pharmacist owners or employee pharmacists) and geographical locations (rural or urban).^30^ Participants were eligible if they were registered health care providers in NL, had provided clinical care for community-based patients and self-reported having taken active steps to integrate deprescribing into their daily practice. All eligible participants were invited to join and were confirmed into the study in the order they accepted. Participants received identical background information about the research, provided informed consent and received a $200 gift card after completing the focus group. Ethics was approved by the Memorial University of Newfoundland Human Research Ethics Board (reference No. 2020.040).

### Data collection

Focus groups consisting of 4 to 5 participants were conducted during November and December 2020 on the Zoom video-conferencing platform to facilitate the attendance of participants from across NL and to comply with COVID-19 requirements. Prior to the focus groups, participants met individually with a researcher online to check the technical setup and ensure familiarity with Zoom. Each focus group was led by an experienced facilitator (J.P.T.), while 3 researchers provided technical support and wrote field notes (D.K., T.S., K.N.). Demographic information, including years in practice (0-4, 5-9, 10-14 or 15+ years), geographical location (urban or rural) and gender, was collected from participants through Zoom’s confidential polling feature. Focus groups were audio and video recorded using Zoom. The audio recordings were transcribed verbatim using Otter software and manually cross-checked for accuracy before being uploaded to NVivo 1.3 qualitative software.

**Figure 1:**
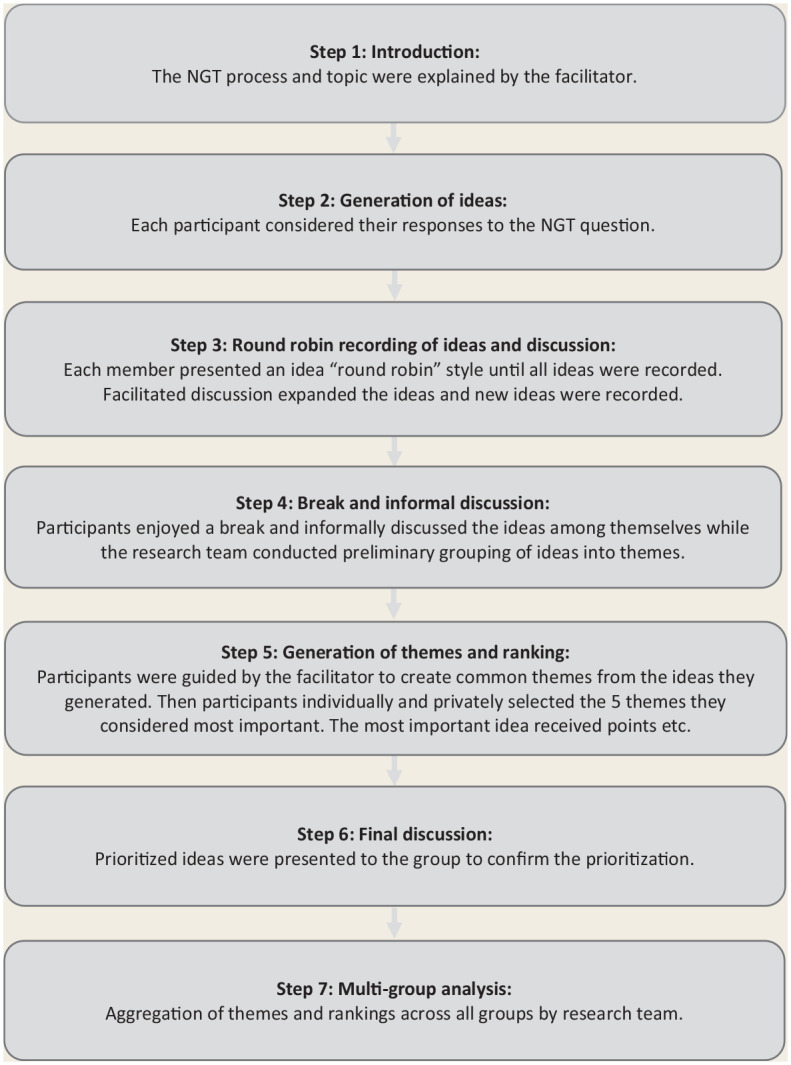
Nominal group technique process

Data collection was guided by Nominal Group Technique (NGT)^29,31^ to elicit group priorities to the following question: “What actions support the implementation of deprescribing into the daily workflow of your practice?” A scripted introduction was read at the start of each focus group to ensure all participants received the same background information and understood the 6-step NGT process.^31^ Each NGT session was then conducted using the same 6-step process: (1) introduction, (2) silent generation of ideas, (3) discussion, (4) generation of themes, (5) ranking of factors and (6) confirmation of results (Figure 1).

### Analysis

Data analysis was conducted in 2 ways: first, through NGT prioritization during the focus group sessions,^31^ and second, by iterative thematic analysis of transcripts to identify themes across focus groups.^32^ During step 5 of the NGT, participants were required to individually and privately select the 5 themes they considered most important from the themes the group generated.^31^ The theme with the higher priority themes was assigned 5 points (4 points for second-highest priority, etc.). The group’s scores were then tallied to produce a list of categories ranked at individual and group levels. To enable comparison across groups (a multigroup analysis),^33^ 1 researcher (T.S.) aggregated group lists by combining duplicate ideas, consulting transcripts for comparison and clarification when necessary and leaving categories separate when possible. In cases in which participant votes were doubled as a result of merging similar categories, the lesser score and extra votes were removed and adjusted results were reported, as recommended for multiple NGT groups.^33^ The resulting single list of categories was discussed and confirmed by the research team.

Audio recordings of the NGT groups were transcribed verbatim to allow thematic analysis to further investigate the themes and to identify patterns across groups. Therefore, the a priori intention was not to collect data until saturation nor return transcripts to participants. One author (T.S.) experienced in thematic analysis inductively coded the full transcripts, identifying patterns through a recursive approach.^32^ The process of thematic analysis also provided an opportunity to cross-check the multiple-group analysis. Because similar categorical names may have different intended meanings between groups, adding thematic analysis improved the rigour and interpretation of the multigroup analysis.^33^ When required to provide additional contexts, the results of the thematic analysis were included in the definitions of the prioritized actions below. This study has been reported in accordance with the Consolidated Criteria for Reporting Qualitative Research (COREQ).^34^

## Results

Five focus groups were held involving pharmacists (*n* = 11), physicians (*n* = 7) and nurse practitioners (*n* = 4). Participants worked in rural (*n* = 10) and urban (*n* = 12) practice settings (Table 1). No participant dropped out or withdrew. Four groups were multidisciplinary in composition, while 1 group consisted of only pharmacists.

**Table 1:** Participant characteristics

Health care provider type	Participants (*N* = 22)	Self-identified gender	Years in practice	Urban or rural practice
Physicians	7	Women (100%)	4 (0-4)	5 urban
			2 (5-9)	2 rural
			1 (10-14)	
			0 (15+)	
Nurse practitioners	4	Women (100%)	1 (0-4)	2 urban
			1 (5-9)	2 rural
			1 (10-14)	
			1 (15+)	
Pharmacist owner	7	4 women (57%)	2 (0-4)	3 urban
		3 men (43%)	2 (5-9)	4 rural
			0 (10-14)	
			3 (15+)	
Pharmacists	4	Women (100%)	3 (0-4)	2 urban
			0 (5-9)	2 rural
			1 (10-14)	
			0 (15+)	
Total	*N* = 22	19 women (86%)	45.5% (0-4)	54.5% urban
		3 men (14%)	22.7% (5-9)	45.5% rural
			13.6% (10-14)	
			18.2% (15+)	

The top 10 actions that participants identified as supporting the implementation of deprescribing into their workflow are listed in Table 2. There was high agreement among the different health care providers and groups, with the top 5 accounting for 68% of the groups’ prioritized scores. The top 5 actions were as follows.

**Table 2:** Top 10 actions that supported the implementation of deprescribing in community practice

Rank	Actions identified and prioritized by participants	Description of action	Adjusted combined scores	Number of groups who listed action	Number of participants who voted for action
1	Patient education	Providing written and verbal patient education	57	5	17
2	Time and resources	Dedicating time and human resources more efficiently and strategically	51	5	14
3	Provider collaboration and communication	Engaging in interprofessional collaboration and communication with providers with shared patients	47	5	16
4	Patient relationships	Building positive and trusting relationships between providers and patients	24	2	7
5	Public awareness strategies	Province-wide public awareness media campaign	22	5	6
6	Alternative treatment	Recommending safer and equally effective drug therapy and nonpharmacologic therapy	17	2	5
7	Provider awareness and education	Participating in continuing education and utilizing deprescribing tools and patient brochures	13	2	6
8	Electronic tools	Utilizing software and programs (HEALTHe NL,* EMR,^†^ pharmacy management software) for reminder alerts	11	2	6
9	Planned and organized workflow	Improving workflow efficiencies and minimizing disruptors	11	1	3
10	Patient identification	Identifying patients who may benefit from deprescribing	10	2	3

* HEALTHe NL is the provincial electronic health record (EHR) for Newfoundland and Labrador (NL).

† eDOCSNL is the provincial electronic medical record (EMR) for NL.

### Providing patient education

Participants prioritized the provision of patient education as the most important action that supported the implementation of deprescribing into their workflow. Health care providers identified that their efforts to implement deprescribing were supported by providing patients with both verbal and written education, such as the SaferMedsNL patient education brochure. Additional actions that facilitated health care providers in providing patient education included interrupting routine refills, receiving alerts in the electronic medical record (EMR), placing SaferMedsNL posters in pharmacies and waiting rooms and providing education about nonpharmacologic alternatives.

Thematic analysis summarized that patient education was an information delivery and patient conditioning process and part of a culture change that focused on shared decision-making and patient values and challenged societal and generational norms.

### Allocating time and resources

Allocating dedicated time and resources was the second-highest ranked action. Participants noted that when they allocated dedicated time for deprescribing within their day, they were increasingly able to engage with patients and other health care providers, which then, in turn, produced additional opportunities for patient-centred and collaborative deprescribing. Allocating time and resources was supported by scheduling dedicated appointment times, investing in extra staff, staff training, help from students and using software to automatically alert if a patient was potentially eligible for deprescribing.

### Building interprofessional collaboration and communication

Nurturing health care provider collaboration and communication ranked as the third most important action that supported the implementation of deprescribing in their practice. Participants reported that both meeting with other health care professionals and openly discussing their individual roles in deprescribing and their preferred methods of communication were vital to deprescribing success. Sharing information and providing consistent messaging to patients increased patient engagement. Participants stated that pharmacists’ relationships with patients and prescribers are an important resource that can be leveraged to promote patients’ changing their medication use behaviour and suggested pharmacists are in a key position to drive medication appropriateness in NL.

### Fostering patient relationships

The fourth highest-ranking action was building patient relationships. Positive provider-patient relationships helped participants facilitate patient engagement in deprescribing. Building trust, collaborating with other health care providers and getting to know patients and understanding their treatment goals were considered extremely important. In addition, following up with patients, personalizing patient education and treatment alternatives and a process of shared decision-making so patients are part of the treatment plan were noted as actions that supported deprescribing in community practice.

Thematically, establishing patient relationships before suggesting changes to medications helped providers build patient trust in deprescribing and led a patient-centred process to facilitate patient engagement and foster positive change.

### Aligning efforts with public awareness campaign

Participants identified that some patients raised the topic of deprescribing and mentioned they had seen the public awareness campaign. Participants described low resistance to the idea of deprescribing as a result of the campaign. Some participants instigated actions to build on the public awareness campaign by using SaferMedsNL brochures and posters within their pharmacy or clinic. Inspired by the public awareness campaign conducted by SaferMedsNL, 1 participant launched their own aligned social media campaign to invite her patients to come in and discuss their reflux medications. Participants highlighted that the public awareness campaign legitimized deprescribing and created opportunities to reinforce patient education and empower patients to start conversations. The public awareness campaign was perceived to support the cultural change necessary to improve appropriate medication use, increase awareness of the merits of deprescribing PPIs and sedatives and reduce the strain on providers to come up with campaigns to engage patients.

## Discussion

Pharmacists, physicians and nurse practitioners identified numerous actions that supported the implementation of deprescribing into daily practice. The top 5 actions were providing patient education, allocating time and resources, nurturing interprofessional collaboration and communication, fostering patient relationships and aligning efforts with public awareness strategies.

Patient-centred education has been shown to improve collaborative deprescribing interventions.^35,36^ In contrast, a recent systematic review highlighted the existence of a “pill for every ill” culture, whereby both patients and health care providers felt like there was an expectation for medications to be prescribed. A recent systematic review of barriers and enablers to deprescribing in primary care identified this culture as a barrier to deprescribing from both patients’ and health care providers’ perspectives.^24^ In contrast, health care providers in NL described the provision of patient education and the SaferMedsNL public awareness campaign as driving a cultural shift across the province in favour of deprescribing. Research in North America has identified that different communication approaches can drive deprescribing behaviour change conversations in primary care, which aligns with our findings.^37-39^ Although patient brochures and deprescribing guidelines^40,41^ have been created to address patient and provider barriers,^28^ a scoping review found that in isolation, addressing just 1 barrier might not be enough.^42^ Future analysis will investigate if SaferMedsNL was able to reduce PPI and sedative use across the province by changing culture and overcoming patient and provider barriers.

In busy clinical settings with numerous competing priorities, allocating resources at a practice level, either by delegating tasks, scheduling specific appointments outside the regular workflow or proactively adding structured prompts in the regular workflow, created a more reliable implementation strategy than solely relying on health care provider awareness to identify potential deprescribing opportunities patients and deliver patient care services. Similar findings were identified in primary care practices in Denmark, where practice-level interventions, including preemptive electronic medical record audits and prescription renewal interventions, supported the allocation of time to deprescribe.^43^ In addition, community pharmacies in Ontario, Canada, reported that overlapping staff rosters enabled dedicated time for deprescribing interventions to be conducted.^44^ Interrupting the routine nature of renewing and refilling prescriptions was an important deprescribing strategy for both pharmacists and prescribers. Health care providers fall into rehearsed prescribing patterns, unconsciously managing time and streamlining clinical decision-making based on familiarity and experience with certain medications.^45^ Placing prompts in health care providers’ environments to divert from routine patterns of behaviour has been demonstrated as a useful technique for interrupting these behaviours.^36^ Similarly, pharmacists highlighted the value of automated alerts in the dispensary computer to identify patients who may be suitable for deprescribing.

Many prescribers acknowledged that electronic prompts might be effective in motivating providers to deprescribe; however, evidence supporting EMR prompts as a means of reducing inappropriate medications is mixed, with some studies showing EMR prompts can reduce prescribing rates of inappropriate medications while others found there was no difference in prescribing rates.^46^ To identify the successful components of deprescribing interventions in primary care, a recent review mapped the intervention components to the behaviour change wheel.^42^ It was observed that barriers to deprescribing were reduced when patients were empowered to initiate the deprescribing process. Likewise, participants prioritized deprescribing when patients initiated the conversation. This approach helped mitigate the burden of identifying potential candidates for deprescribing. Participants felt patient brochures and clinical guidelines supported their capacity and confidence to lead deprescribing in their practice.

Interdisciplinary collaboration and communication are key strategies to optimize medication management.^35^ Improving interprofessional communication has been shown to increase prescriber acceptance of collaborative patient-centred deprescribing.^47,48^ Patients see multiple health care providers often with little shared communication, resulting in fragmentation of care.^49^ This can contribute to a repetitive cycle of prescribing and a challenge for deprescribing. Although participants identified system-level information and communication barriers, several in-practice changes were made to mitigate the barriers posed by fragmentation communication. Findings suggest building interprofessional relationships and creating a culture of medication appropriateness can help mitigate some of the real-world barriers reported by community pharmacists when deprescribing, such as prescriber engagement, role uncertainty and information barriers.^30^ This is consistent with a systematic review of implementation, which identified the most commonly reported contextual features that influence the implementation of evidence-based activities in health care are organizational culture, resources, communication, collaboration and leadership.^50^

Fostering patient relationships was noted to improve deprescribing by establishing a patient-centred process to facilitate engagement. Consistent with other research, participants identified the importance of establishing a trusting relationship with patients to facilitate deprescribing.^51,52^ A qualitative study including community-dwelling older adults in Australia identified that prescribers need to foster patient relationships and adapt their communication about polypharmacy based on their patients’ attitudes to medicines and preferences for involvement in decisions.^53^ Similarly, participants in this study noted that changing expectations around refills and modelling medication appropriateness by promoting prudent prescribing practices improved patient relationships. Although some participants alleged that creating boundaries risked damaging patient relationships, others described that establishing prescription expectations strengthened their relationship by educating patients who did not realize there could be a reason for concern. It is difficult to determine which patients will respond positively to the idea of deprescribing,^54^ therefore, deprescribing conversations should take place with all patients.

The public awareness campaign to promote deprescribing was highly valued by participants because it was seen to normalize the idea of deprescribing PPIs and sedatives. The public awareness campaign promoted patient engagement in deprescribing and reduced the burden placed on providers to initiate the topic of deprescribing. Participants’ descriptions of the PPI and sedative awareness campaigns align with descriptions of antibiotic awareness campaigns that have led to improved antibiotic use.^55^ A review of public awareness campaigns promoting appropriate antibiotic use identified that successful campaigns targeted both patients and health care providers, as did the SaferMedsNL campaign. A review of public engagement on deprescribing conducted by the Canadian Medication Appropriateness and Deprescribing Network stated that activities to promote public awareness should be prioritized.^56^ This was based on a pan-Canadian survey of 2665 community-dwelling seniors that found that respondents were 75% more likely to initiate deprescribing conversations when they were aware that certain medications could be harmful.^57^ Future research will assess whether or not the public awareness campaigns drove people to the SaferMedsNL.ca website; however, until those results are available, the results of this study suggest the public awareness campaign was successful in encouraging patients to become more involved in their medications from the health care providers’ perspective.

This research was conducted to identify and share strategies found to support the implementation of deprescribing, to assist health care providers in entrenching deprescribing into their daily practice. Previously identified barriers to implementing deprescribing in long-term care facilities included a lack of system configurations and funding models that limited providers’ ability to spend time with patients and restricted provider communication and collaboration.^22,58^ Poor remuneration has been reported as a barrier to deprescribing by family physicians and pharmacists in Australia^22^ and Germany,^30^ while community pharmacists in Canada and Germany have reported a perverse disincentive, with deprescribing leading to financial losses.^30,59^ While several participants in this study reported a similar perspective, others discussed how they were able to implement small changes within their practice to improve collaboration, regardless of financial impact. Others discussed making a deliberate effort to talk to other prescribers and pharmacists about deprescribing and understanding each provider’s roles and expectations. Having clear roles and building these professional relationships allowed them to integrate patient-centred deprescribing more effectively. Having multiple providers in a patient’s circle of care who promote deprescribing can influence patients’ acceptance of deprescribing, as outlined in a scoping review of deprescribing interventions in primary care.^42^ In 22 of the 43 identified articles, the domain of “social support” was attributed to supporting deprescribing, with pharmacists and physicians working together to conduct medication reviews, identifying inappropriate medications and collaboratively developing the deprescribing process. Further research may be required to better understand how health care providers’ perceptions of time constraints may differ by remuneration models and providers. Understanding the prompts that are most effective in promoting health care provider engagement is also an important direction for further study, particularly for collaborative practice.

This study had many strengths, including the combined NGT with thematic analysis. The utilization of a virtual platform improved accessibility and enabled province-wide recruitment and participation of community pharmacists, physicians and nurse practitioners, which maximized the variation of observations and experiences. To reduce recruitment bias, we used a purposeful sampling framework to respect that health care providers working in different contexts (roles or locations) may have different perspectives. Furthermore, recruitment was widely promoted through social media and key health professional bodies from NL. Despite this, there remains a possibility of recruitment bias because only health care providers who were actively engaged in deprescribing responded. However, this was considered acceptable because the aim was to identify which actions were producing positive uptake of deprescribing. The stratified sampling framework tried to encourage the inclusion of different participant perspectives; however, we were constricted in recruiting participants who chose to respond to the advertising. Because the advertising was promoted by external stakeholders, we do not know how many health care providers saw the advertising, and consequently, it was not possible to determine the response rate. As the study enrolled participants only from NL, the findings may not be generalizable to other Canadian provinces or internationally. Although data saturation was not a goal, thematic analysis identified consistent themes between groups, with no new themes emerging within the last group.

## Conclusion

This study identified actions that support the implementation of deprescribing in day-to-day practice for various community-based providers. Despite different practice settings and professional roles, participants identified similar supportive actions, with 5 being voted most important: (1) providing patient education, (2) allocating time and resources, (3) building interprofessional collaboration and communication, (4) fostering patient relationships and (5) aligning with public awareness strategies. Sharing these supportive implementation actions may help other health care providers embed deprescribing into their daily practice. ■
